# Meta-analysis of pharmacogenetic interactions in amyotrophic lateral sclerosis clinical trials

**DOI:** 10.1212/WNL.0000000000004606

**Published:** 2017-10-31

**Authors:** Ruben P.A. van Eijk, Ashley R. Jones, William Sproviero, Aleksey Shatunov, Pamela J. Shaw, P. Nigel Leigh, Carolyn A. Young, Christopher E. Shaw, Gabriele Mora, Jessica Mandrioli, Giuseppe Borghero, Paolo Volanti, Frank P. Diekstra, Wouter van Rheenen, Esther Verstraete, Marinus J.C. Eijkemans, Jan H. Veldink, Adriano Chio, Ammar Al-Chalabi, Leonard H. van den Berg, Michael A. van Es, For UKMND-LiCALS and LITALS Study Group

**Affiliations:** From the Department of Neurology, Brain Centre Rudolf Magnus (R.P.A.v.E., F.P.D., W.v.R., J.H.V., L.H.v.d.B., M.A.v.E.), and Department of Biostatistics and Research Support (M.J.C.E.), University Medical Centre Utrecht, the Netherlands; Maurice Wohl Clinical Neuroscience Institute and United Kingdom Dementia Research Institute Centre (A.R.J., W.S., A.S., C.E.S., A.A.-C.), Department of Basic and Clinical Neuroscience, King's College London; Sheffield Institute for Translational Neuroscience (SITraN) (P.J.S.), University of Sheffield, South Yorkshire; Department of Clinical Neuroscience (P.N.L.), Trafford Centre for Biomedical Research, Brighton and Sussex Medical School, Falmer, Brighton; The Walton Centre NHS Trust (C.A.Y.), Liverpool, UK; Istituti Clinici Scientifici Maugeri IRCSS (G.M.), Milan; Department of Neuroscience (J.M.), Sant'Agostino-Estense Hospital and University of Modena and Reggio Emilia, Modena; Department of Neurology (G.B.), Azienda Universitario Ospedaliera di Cagliari and University of Cagliari; Istituti Clinici Scientifici Maugeri IRCSS (P.V.), Mistretta, Italy; Rijnstate Ziekenhuis (E.V.), Arnhem, the Netherlands; Rita Levi Montalcini' Department of Neuroscience (A.C.), ALS Centre, University of Torino; and Azienda Ospedaliera Città della Salute e della Scienza (A.C.), Turin, Italy.

## Abstract

**Objective:**

To assess whether genetic subgroups in recent amyotrophic lateral sclerosis (ALS) trials responded to treatment with lithium carbonate, but that the treatment effect was lost in a large cohort of nonresponders.

**Methods:**

Individual participant data were obtained from 3 randomized trials investigating the efficacy of lithium carbonate. We matched clinical data with data regarding the *UNC13A* and *C9orf72* genotype. Our primary outcome was survival at 12 months. On an exploratory basis, we assessed whether the effect of lithium depended on the genotype.

**Results:**

Clinical data were available for 518 of the 606 participants. Overall, treatment with lithium carbonate did not improve 12-month survival (hazard ratio [HR] 1.0, 95% confidence interval [CI] 0.7–1.4; *p* = 0.96). Both the *UNC13A* and *C9orf72* genotype were independent predictors of survival (HR 2.4, 95% CI 1.3–4.3; *p* = 0.006 and HR 2.5, 95% CI 1.1–5.2; *p* = 0.032, respectively). The effect of lithium was different for *UNC13A* carriers (*p* = 0.027), but not for *C9orf72* carriers (*p* = 0.22). The 12-month survival probability for *UNC13A* carriers treated with lithium carbonate improved from 40.1% (95% CI 23.2–69.1) to 69.7% (95% CI 50.4–96.3).

**Conclusions:**

This study incorporated genetic data into past ALS trials to determine treatment effects in a genetic post hoc analysis. Our results suggest that we should reorient our strategies toward finding treatments for ALS, start focusing on genotype-targeted treatments, and standardize genotyping in order to optimize randomization and analysis for future clinical trials.

Despite considerable efforts, riluzole is still the only drug that has been shown to increase survival in patients with amyotrophic lateral sclerosis (ALS).^1^ Phenotypic, genetic, and pathophysiologic heterogeneity form a plausible explanation for the large number of negative trials in ALS.^2^ Although the mechanisms underlying ALS are not fully understood,^2^ it is clear that genetic variation plays an important role in both familial and sporadic ALS.^3^ It is reasonable to hypothesize that mutations in many different genes may act through several different pathways, but that they all cause motor neurodegeneration and manifest with an ALS phenotype. It may, therefore, be conceivable that different subtypes of ALS respond differently to disease-modifying therapies and multiple individually tailored therapies may need to be developed to treat the disease effectively.

Within the field of oncology, the treatment for a specific type of malignancy often depends on the genetic tumor characteristics. For instance, patients with melanoma and *BRAF* gene mutations have significantly improved rates of overall and progression-free survival when treated with a BRAF kinase inhibitor.^4^ It seems that therapeutic strategies for ALS are also moving toward precision medicine and groundbreaking targeted trials for *SOD1*-related ALS have already been undertaken or are underway with antisense oligonucleotides,^5^ arimoclomol (ClinicalTrials.gov NCT00706147), and pyrimethamine.^6^

In this study, we explore the possibility that patients with genetic subgroups of ALS may have responded to treatment in previously conducted negative trials evaluating lithium carbonate, but that a proportionally larger cohort of nonresponders diluted the treatment effect in the overall analysis.

## METHODS

### Study design.

When performing post hoc analyses according to genotype and re-estimating treatment effects for genetic subgroups, it is important to recognize that several problems will arise. First, the sample size within each subgroup will decrease dramatically and statistical power to detect treatment differences is severely reduced. Second, obtaining DNA samples and genetic screening is not standard practice in ALS clinical trials, thus one can expect that genetic data will be missing. Finally, over 30 genes have been associated with ALS. This may further reduce the statistical power by multiple testing, but more importantly, will inflate the false-positive risk. To overcome these issues, we performed an individual participant data (IPD) meta-analysis of randomized controlled trials with lithium carbonate in ALS; multiple trials with this compound have been performed and, therefore, a large sample size could be obtained. Moreover, an IPD meta-analysis enabled us to reduce the false-positive risk by validating trends in independent cohorts of patients and improve generalizability. Genetic post hoc analyses were limited to (1) genes in which variation is relatively common in order to ensure sufficient numbers and (2) genes known to be modifiers of prognosis. We therefore included 2 genetic subgroups: (1) *C9orf72* repeat expansion carriers and (2) patients homozygous for the C allele of rs12608932 located in *UNC13A*. Repeat expansions in *C9orf72* are the most common genetic cause of ALS and are found in approximately 5%–10% of patients with ALS of European descent (familial and sporadic cases combined).^7,8^ Genome-wide association studies (GWAS) have repeatedly detected an association for a single nucleotide polymorphism (SNP: rs12608932) located in the *UNC13A* gene.^9–11^ The effect of this SNP on disease risk is modest, with an odds ratio <1.30, but appears to convey a large effect on survival. Multiple studies have shown that the mean survival in patients homozygous for the C allele of rs12608932 is 6 to 12 months shorter, implying that this SNP, or variants in linkage disequilibrium with it, is a strong phenotypic modifier and therefore of biological relevance.^12–15^ Approximately 16% of patients with ALS are homozygous for the C allele of rs12608932.^9–11^

### Search strategy and study selection.

To identify randomized clinical trials evaluating the efficacy of lithium carbonate in patients with ALS, we systematically searched the PubMed database, Embase, Cochrane Library, Web of Science, and online clinical trial registers (ClinicalTrials.gov, EudraCT, and IRCTN) up to November 2016. The following search terms were used: “amyotrophic lateral sclerosis” or “motor neuron* disease” or “Lou Gehrig*,” and “lithium*.” Reference lists from relevant reviews and included trials were screened in order to retrieve additional studies. Only clinical trials published in English were included. Each study was assessed for its methodologic quality and risk of bias for confounding, detection, performance, attrition, and reporting bias.^16^ We included only randomized clinical trials with an overall low risk of bias; see table e-1 at Neurology.org for the scoring of the included studies. We identified 4 clinical trials that provided a minor risk of bias and subsequently contacted the relevant corresponding authors for the individual participant and genotypic data (figure e-1). Three groups (the Netherlands, United Kingdom, and Italy) agreed to participate in the IPD meta-analysis with genetic post hoc analyses.

### Standard protocol approvals, registrations, and patient consents.

The initial trials were all conducted according to the International Conference on Harmonisation Good Clinical Practice guidelines and with the approval of local ethical and institutional review boards. All informed consents permitted the use of IPD for future post hoc analyses, but did not specifically state genetic post hoc analyses. We therefore obtained permission from local ethical and institutional review boards to use existing genotype data from genetic studies in which trial participants were simultaneously enrolled, or to genotype DNA samples if available. This meant that the trials and genetic studies had to be temporarily deanonymized in order to match clinical data to genetic data or DNA samples. After linking these files, the data were reanonymized.

### Genotyping of DNA samples.

For all samples, *C9orf72* had either been genotyped previously or was genotyped after obtaining a DNA sample using repeat-primed PCR as described previously.^17^ The majority (64%) of the available DNA samples from trial participants has been included in previously conducted GWAS using Illumina (San Diego, CA) BeadChips and provided genotype data for rs12608932. In the remaining samples (36%), this SNP was genotyped using Taqman (Applied Biosystems, Foster City, CA) assays, as described previously.^18^

### Definitions and outcome measures.

Based on previous literature, patients with the *UNC13A* C/C genotype were classified as *UNC13A* carriers in the subsequent analyses; the remaining patients with the *UNC13A* A/C or A/A genotype were classified as noncarriers.^14^ Patients with more than 30 repeats in the *C9orf72* gene were considered to be *C9orf72* carriers.^19^ Our a priori primary measure of treatment efficacy was death from any cause at 12 months after randomization. Due to the high adverse event and nonadherence rate, setting the follow-up to 12 months was thought to best capture a possible therapeutic effect and minimize the risk of diluting the effect by the intention-to-treat principle of analysis.

### Statistical analysis.

All outcomes were analyzed according to the intention-to-treat principle of analysis. We chose to analyze the IPD from the 3 trials using a one-step meta-analytic approach. Previous studies have shown that a one-step meta-analytic approach provides similar treatment effect estimates, if clustering is appropriately accounted for, in comparison with a 2-step approach (e.g., first summarizing the individual trial data [step 1], before pooling the effect estimates [step 2]).^20^ The IPD from the 3 studies were merged together and a study indicator variable was created. We performed a pooled analysis, while adjusting for the clustering within studies by stratifying each analysis for the study indicator. Missing data in covariates (1.5% of the cases had at least one missing value) did not predict the outcome (*p* = 0.50); therefore, all missing values in the covariates, except for the genetic data, were imputed with their mean. Unlike in observational studies, mean imputation has been shown to give unbiased estimates of the treatment effect in randomized controlled trials.^21^ When we analyzed genetic interactions with lithium carbonate, we used only patients with complete genetic data, as phenotypic variables were unable to predict the genotype accurately. We prespecified one sensitivity analysis by estimating the treatment effect with and without the control group of Chio et al.,^22^ as this control group used a subtherapeutic dose of lithium (0.2–0.4 mEq/L instead of 0.4–0.8 mEq/L).

The time to event outcome was analyzed using Cox proportional hazard models, stratified by the study indicator. Adjustment for prognostic covariates substantially increases the statistical power of Cox proportional hazard models.^23^ Therefore, we selected the most important predictors by stepwise backward selection using Akaike Information Criterion. The selected predictors were subsequently added to the model. Next, the treatment indicator variable (lithium or control) was incorporated in the model. The difference in log likelihoods between a model with and without the treatment variable was calculated and significance testing was done by the likelihood ratio test. Using the same testing procedure, we evaluated whether the treatment effect depended on the *C9orf72* or *UNC13A* genotype by incorporating 2-way interaction terms. Due to the exploratory, nonconfirmatory nature of this genetic post hoc subgroup analysis, we did not correct significance levels for multiple testing. Results were considered significant when the 2-sided *p* value was lower than 0.05.

## RESULTS

Data were available for 518 participants in 3 randomized clinical trials evaluating the efficacy of lithium carbonate; study characteristics are given in table 1. Individual data were not available from 1 of the 4 clinical trials (study by Aggarwal et al.^24^), which involved 88 participants. Baseline characteristics of the participants included in the analysis are given in table 2. Complete data regarding the main prognostic confounders were available for 98.5% of the participants (8 patients had an unknown date of onset). In total, 261 (50.4%) patients received lithium carbonate and 257 (49.6%) patients were allocated to the control arm, in which 174 patients received placebo (67.4%) and 83 patients a subtherapeutic dose of lithium carbonate (32.3%). The baseline characteristics were well-balanced between the lithium carbonate and control groups.

**Table 1:**
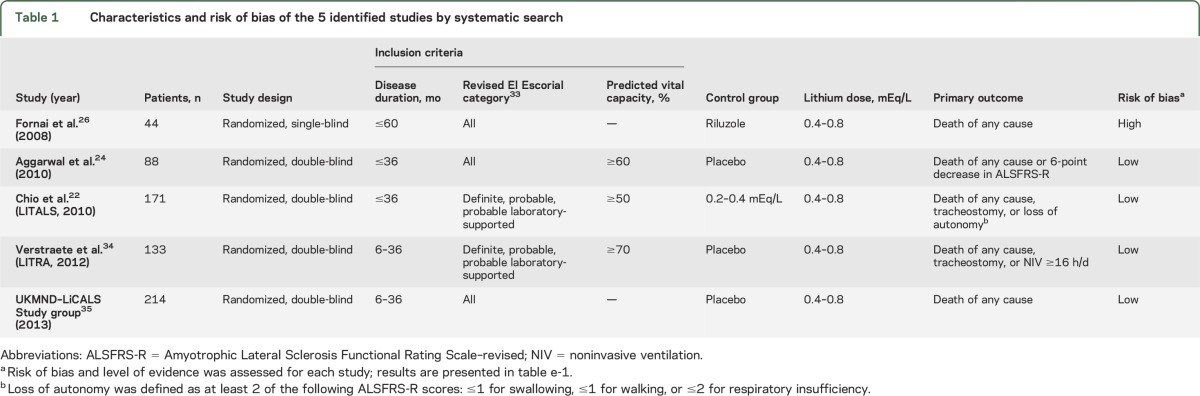
Characteristics and risk of bias of the 5 identified studies by systematic search

**Table 2:**
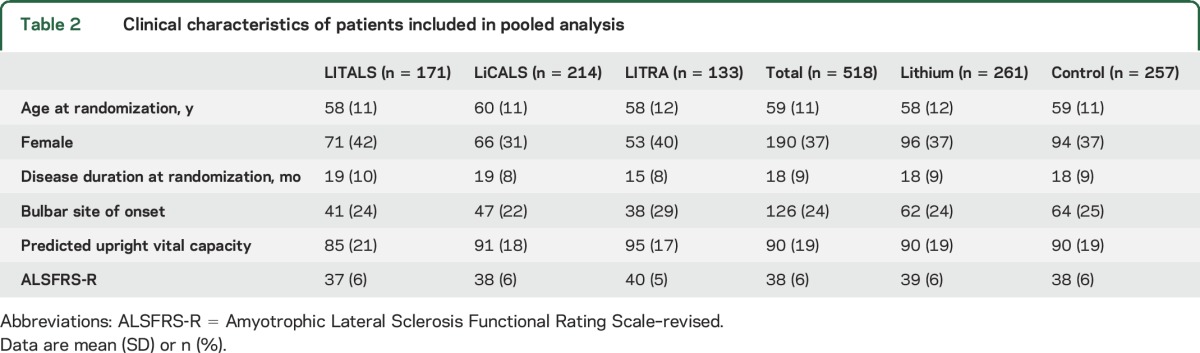
Clinical characteristics of patients included in pooled analysis

**Figure 1:**
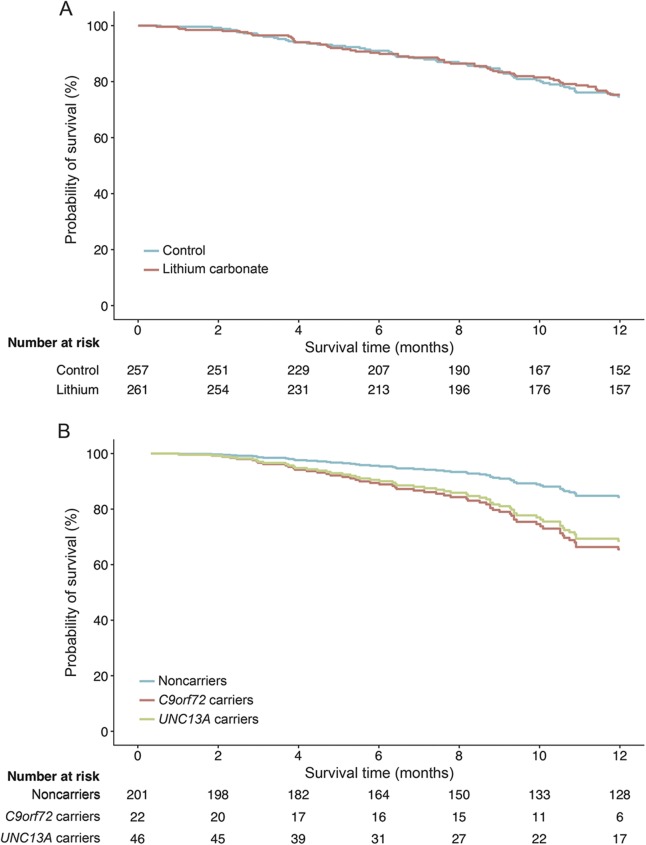
Pooled analysis of treatment effect for lithium carbonate and 12-month survival for each genetic subgroup Pooled 12-month survival in 3 clinical trials evaluating the efficacy of lithium carbonate. (A) Overall treatment effect of lithium carbonate was nonsignificant (hazard ratio [HR] 1.0, 95% confidence interval [CI] 0.7–1.4). (B) There was a significant effect of genetic subgroups on 12-month survival, irrespective of treatment arm, within the clinical trials (*UNC13A* HR 2.4, 95% CI 1.3–4.3; *p* = 0.006; and *C9orf72* HR 2.5, 95% CI 1.1–5.2; *p* = 0.032). Three patients had both risk variants of *UNC13A* and *C9orf72*; the number at risk of these patients is merged with the *UNC13A* carriers.

Age, Amyotrophic Lateral Sclerosis Functional Rating Scale–revised (ALSFRS-R) slope, and vital capacity at baseline were predictors for survival at 12 months (all *p* < 0.001) and were adjusted for in all subsequent analyses (table e-2). Overall, 75.3% (95% confidence interval [CI] 69.9–81.2) of the patients in the control arm and 74.7% (95% CI 69.1–80.6) in the lithium arm were still alive at 12 months, corresponding to an adjusted hazard ratio (HR) of 1.0 (95% CI 0.7–1.4; *p* = 0.96; figure 1A). Excluding the subtherapeutic control group from the analysis did not change the treatment effect (HR 1.3, 95% CI 0.9–2.1; *p* = 0.21). Next, we evaluated the prespecified genetic subgroup interactions in all patients with genetic data (n = 269); the baseline characteristics are given in tables 3 and e-3. Both the *UNC13A* and *C9orf72* genotype were independent predictors for 12-month survival, with an adjusted HR of 2.4 (95% CI 1.3–4.3; *p* = 0.006) and HR 2.5 (95% CI 1.1–5.2; *p* = 0.032), respectively (figure 1B). The overall effect of lithium carbonate in the patients with genetic data remained futile (HR 0.8, 95% CI 0.4–1.4; *p* = 0.39).

**Table 3:**
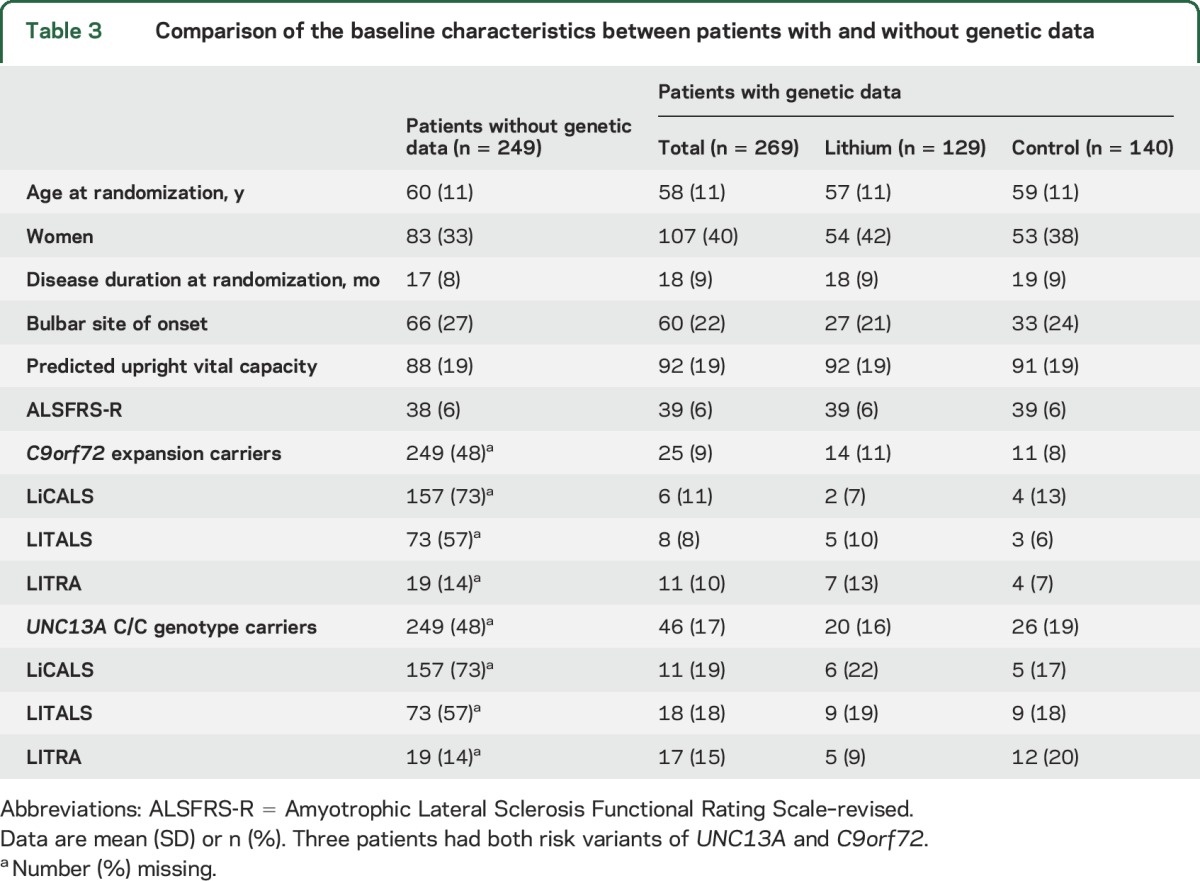
Comparison of the baseline characteristics between patients with and without genetic data

**Figure 2:**
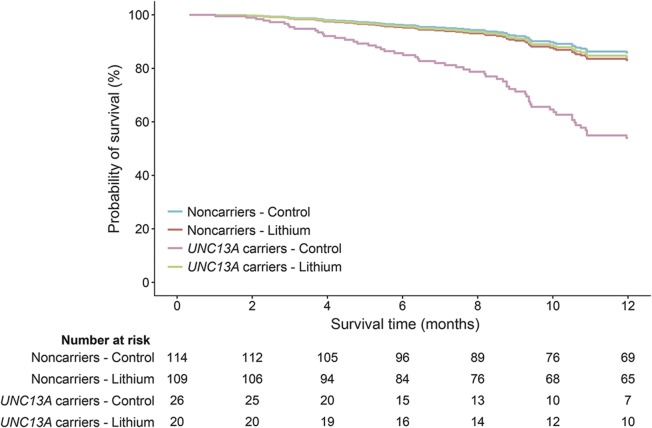
Cox proportional hazards model of 12-month survival and the interaction of lithium carbonate with *UNC13A* genotype Incorporating interaction terms between treatment arm (control or active) and *UNC13A* carrier status revealed that the effect of lithium carbonate significantly depended on the *UNC13A* carrier status (*p* = 0.027). Lithium carbonate improved the 12-month survival in individuals with the *UNC13A C/C* genotype, but had no effect in noncarriers.

The treatment effect was different for the *UNC13A* carriers (n = 46; *p* = 0.027) but not for the *C9orf72* carriers (n = 25; *p* = 0.22). Lithium carbonate in *UNC13A* carriers resulted in a 70% reduction in the number who died during the 12-month follow-up period as compared to the placebo group (HR 0.3, 95% CI 0.1–0.9), whereas the noncarriers did not benefit from lithium carbonate (HR 1.2, 95% CI 0.6–2.3; figure 2). The significant treatment interaction with *UNC13A* genotype remained after correcting for the interaction between the *C9orf72* genotype and lithium (*p* = 0.020) or excluding the control group from the LITALS study (*p* = 0.047). The interaction between lithium treatment and *UNC13A* was homogenous across the 3 different studies (3-way interaction Cox model; *p* = 0.99; figure e-2). Baseline characteristics of the *UNC13A* carriers are given in table e-3 (n = 46). The crude Kaplan-Meier estimate of 12-month survival probability for *UNC13A* carriers improved from 40.1% (95% CI 23.2–69.1) in the control group (n = 26) to 69.7% (95% CI 50.4–96.3) in the lithium group (n = 20) (*p* = 0.056). When we adjusted for baseline inequalities (vital capacity and sex), lithium treatment was effective (*p* = 0.039), and remained so when we additionally corrected for age and ALSFRS-R slope (*p* = 0.040).

## DISCUSSION

In this study, we have shown the importance of including genetic information in clinical trials for ALS. Our results reveal that even within a well-defined and selected trial population, considerable differences in the primary outcome can be expected for patients with either the *UNC13A* C/C genotype or *C9orf72* repeat expansion. Interestingly, we showed that the overall meta-analysis of trials with lithium carbonate in ALS is futile, but that a genetic subgroup of patients (*UNC13A* C/C genotype) may benefit from this treatment. Due to the small sample size of this genetic subgroup (fewer than 20% of the cases), the signal indicating response may have been lost within the large group of nonresponders.

Although our genetic knowledge about causative and disease-modifying genes in ALS is growing exponentially,^3^ we have not yet managed to translate these novel findings into effective therapeutic strategies. To date, only 2 targeted (phase I) genetic trials have been completed and a number of targeted trials are currently underway.^5,6^ By showing that genetic variation in ALS genes significantly influences the primary outcome measure of a clinical trial and may alter treatment response, we have demonstrated the importance of incorporating genetic data in the analysis of ALS trials. Unequally balanced genotypes across treatment and control groups, especially in smaller studies, may greatly influence the false-positive and false-negative rates and the validity of clinical trials in ALS as a whole. For instance, the probability of an imbalance larger than 10% between treatment arms, if the prognostic factor is present in 15% of the cases (like *UNC13A* C/C genotype), is 0.24 and 0.10 for trial sizes of n = 50 and n = 100, respectively.^25^ It might therefore even be conceivable that the high false-positive rate of the phase II trial in ALS^2^ is partially caused by an imbalance of disease-modifying genetic variants between treatment arms in these studies. The false-positive risk may be further inflated by the limited sample size often used for phase II ALS trials.

Lithium for ALS first came into the spotlight after an initial report that suggested an important improvement of survival following lithium treatment.^26^ Our study, combining the results of 3 randomized placebo-controlled trials, excludes an overall treatment effect similar to riluzole. We had 89% power to detect a 10% absolute increase in survival.^27^ We found, however, that the treatment effect of lithium carbonate was not homogenous across patients. The observation that patients with ALS homozygous for the C allele of rs12608932 in *UNC13A* may benefit from lithium may warrant further research. The UNC13A protein is involved in synaptic vesicle maturation and neuronal outgrowth.^28^ Lithium has been shown to influence many pathways, including the induction of sprouting of pyramidal neurons in the corticospinal tract and the promotion of synaptogenesis, and plays a role in autophagy.^29^ All these mechanisms are potentially relevant to ALS. However, it has also been shown that rs12608932 influences the expression of the nearby *KCNN1* gene,^30,31^ which encodes a potassium calcium-activated channel. It is therefore also possible that lithium influences KCCN1 or acts through other pathways.

Without a solid understanding of the biological interaction between the treatment and pathophysiologic pathway, it is challenging to robustly identify the responder group, without increasing the risk of drawing false-positive or false-negative conclusions.^32^ We reduced this likelihood by only testing 2 prespecified pharmacogenetic interactions and selecting genotypes that are relatively commonly occurring in the general ALS population. Moreover, by using data from 3 independent cohorts, we could assess whether the signal is consistent across studies. Nevertheless, the evidence we provide regarding the interaction between *UNC13A* and lithium carbonate is still exploratory and hypothesis-generating. This finding does, however, warrant further exploration of lithium carbonate in a well-balanced, blinded, randomized clinical trial specifically targeted at patients with ALS and the *UNC13A* C/C genotype. Such a trial, and future genetic trials for ALS in general, will require intensive international cooperation to obtain large sample sizes of patients with ALS with a specific genotype. For instance, the prevalence of the *UNC13A* C/C genotype is 12.2%–19.5%^9,12,15^ among patients with ALS. This would result in a screening failure rate of 80.5%–87.8% on genotype alone. Large numbers of patients will need to be approached to ensure an acceptable phase III clinical trial sample size. For instance, 140 *UNC13A* carriers would be required to detect a HR of 0.62 by a 2-sided log-rank test with 90% power, assuming a 1-year survival of 50% in the placebo group, indicating that in the worst case (*UNC13A* prevalence of 12.2%), approximately 1,100 patients need to be genotyped.

ALS is both clinically and genetically a highly heterogeneous disease and it is this complexity that seems to complicate the development of effective treatment for our patients. Even in carefully selected trial populations, the genotype significantly affected the primary outcome measure—survival—in ALS trials. The assumption of a homogenous treatment effect across patients with ALS, for lithium specifically and ALS trials in general, seems no longer tenable and genetic subgroups of patients may modify the treatment effect. The results from this study suggest that we should reorient our strategies toward finding treatments for ALS and start focusing on genotype-targeted treatments and standardize genotyping in order to optimize randomization and analysis in ALS clinical trials.

## Supplementary Material

Data Supplement

Coinvestigators

Accompanying Editorial

## GLOSSARY

ALS: amyotrophic lateral sclerosis
ALSFRS-R: Amyotrophic Lateral Sclerosis Functional Rating Scale–revised
CI: confidence interval
GWAS: genome-wide association studies
HR: hazard ratio
IPD: individual participant data
SNP: single nucleotide polymorphism
