# Supplementary material for: Meta-analysis of pharmacogenetic interactions in amyotrophic lateral sclerosis clinical trials
Source: Neurology. 2017 Oct 31;89(18):1915–22. doi: 10.1212/WNL.0000000000004606 (PMC5664299; doi:10.1212/WNL.0000000000004606)
Supplement: Data Supplement [file supp_WNL.0000000000004606_Supplemental_Material.pdf]

Figure e-1. Flow diagram of study selection.

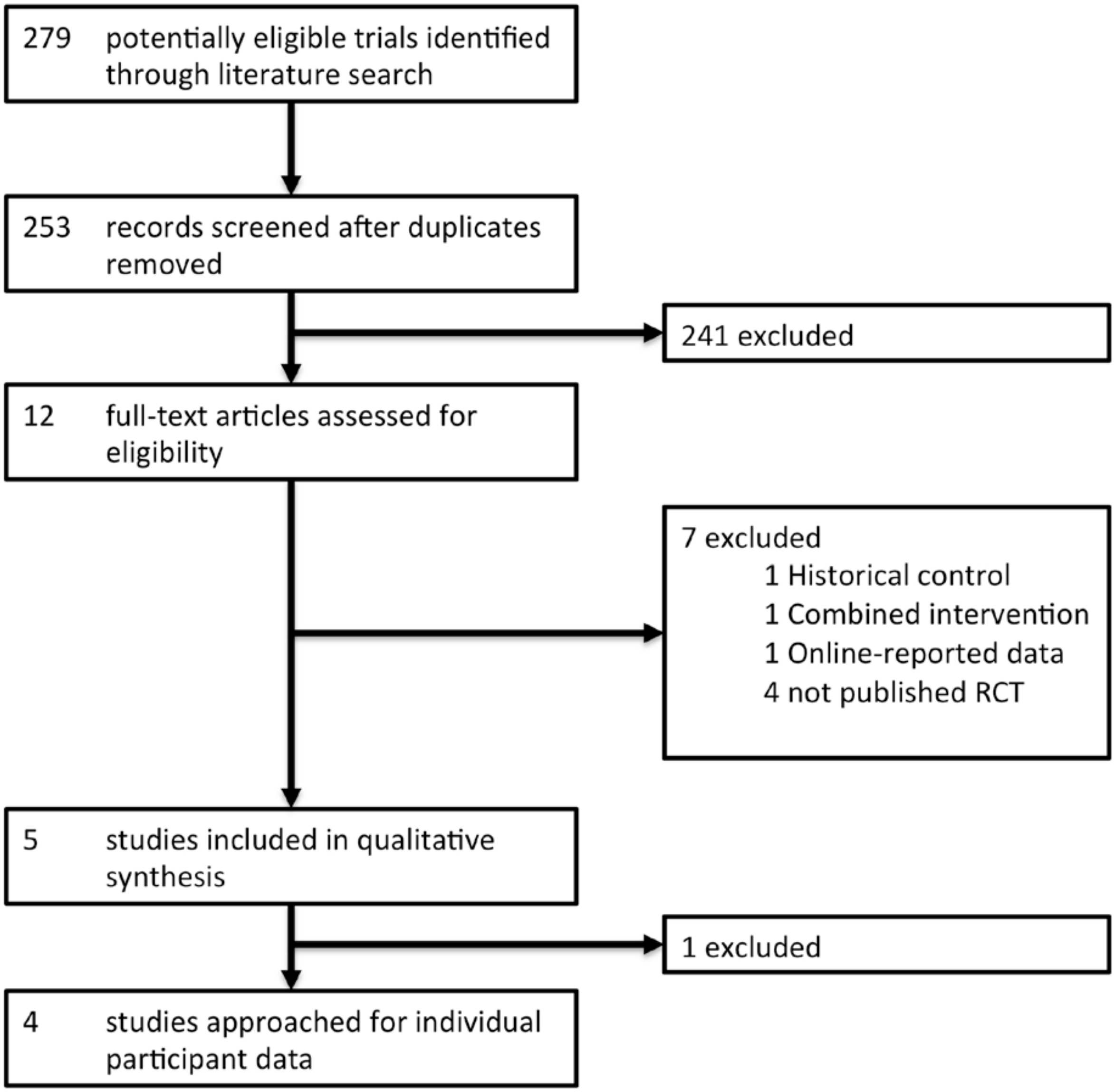

Flow diagram of the systematic search and identification of randomized, controlled clinical trials investigating the efficacy of lithium carbonate.

Figure e-2. Kaplan-Meier curves per trial for UNC13A carriers and the effect of lithium carbonate (n = 46).

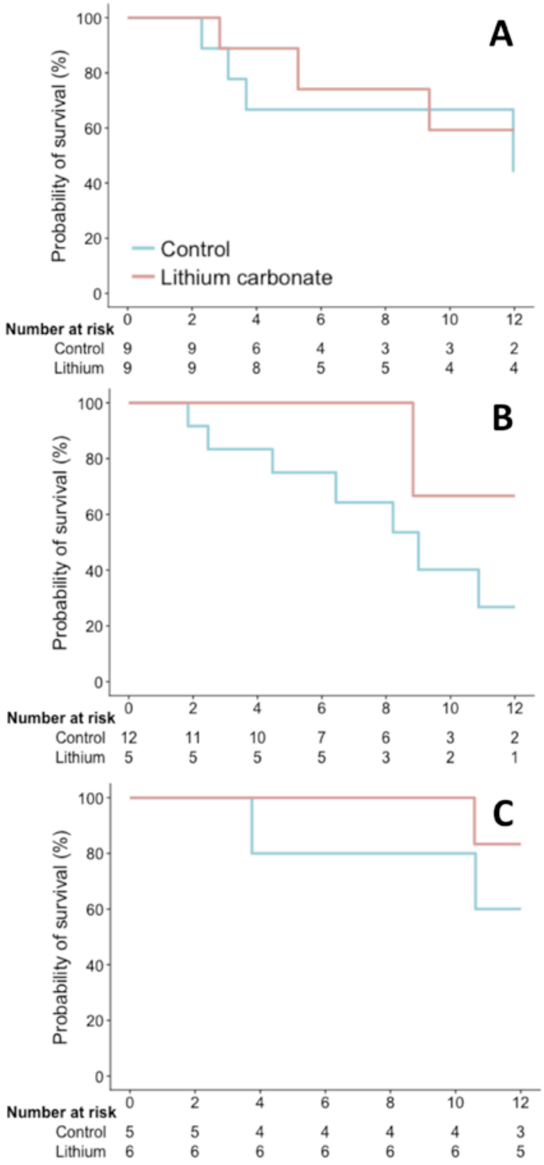

For each trial we plotted the Kaplan-Meier curves for only the UNC13A carriers to assess whether the treatment interaction with the UNC13A depended on the trial. Visually, the effect of lithium seems to be homogenous across trials; every trial shows a similar pattern with an improved survival probability in the lithium group for UNC13A carriers (A = LITALS, B = LITRA, C = LICALS). Incorporating a three-way interaction in the full model between treatment, UNC13A and trial ID also indicated this: the interaction between lithium – UNC13A does not depend on trial ID (p=0.99).

Table e-1*:* Assessment of risk of bias per study.

**Study (year)**	**Confounding**	**Performance bias**	**Detection bias**	**Attrition bias**	**Reporting bias**	**Overall**	**Level of evidence[a]**
Fornai *et al* [1] *(2008)*	*High Risk;* Unknown randomization	*High risk;* Unblinded participants	*Low risk*	*High risk;* Unknown missing data or analysis used	*High risk;* No protocol available	*High risk*	2B; *small sample size*
Aggarwal *et al* [2] *(2010)*	*Low risk*	*Low risk*	*Low risk*	*High risk;* More missing data in lithium group	*Low risk*	*Low risk*	1B
Chio *et al* [3] (2010)	*High risk;* No allocation concealment	*Low risk*	*Low risk*	*High risk;* Missing data	*Low risk*	*Low risk*	2B; *small sample size completers*
Miller *et al* [4] *(2011)*	*High risk;* Non randomized	*High risk;* Unblinded	*High risk;* Unblinded	*Low risk*	*Low risk*	*High risk*	4
Wicks *et al* [5] *(2011)*	*High risk;* Non randomized	*High risk;* Unblinded	*High risk;* Unblinded	*Low risk*	*High risk;* No protocol available	*High risk*	4
Verstraete *et al* [6] *(2012)*	*High Risk;* Unknown randomization	*Low risk*	*Low risk*	*High risk;* More missing data in lithium group	*Low risk*	*Low risk*	1B
UKMND – LiCALS Study group [7] *(2013)*	*Low risk*	*Low risk*	*Low risk*	*Low risk*	*Low risk*	*Low risk*	1B

Confounding = randomization & allocation concealment; Performance = blinding personel and patients; Detection = blinded outcome assessment; Attrition = participants included in analysis and handling missing data; Reporting = protocol available and selective reporting of outcomes.

[a]Level of evidence was based on the scale developed by the Centre for level of evidence, Oxford (www.cebm.net).

References Table e-1

1. Fornai F, Longone P, Cafaro L, et al. Lithium delays progression of amyotrophic lateral sclerosis. Proc Natl Acad Sci U S A 2008;105:2052-2057.

2. Aggarwal SP, Zinman L, Simpson E, et al. Safety and efficacy of lithium in combination with riluzole for treatment of amyotrophic lateral sclerosis: a randomised, double-blind, placebo-controlled trial. The Lancet Neurology 2010;9:481-488.

3. Chio A, Borghero G, Calvo A, et al. Lithium carbonate in amyotrophic lateral sclerosis: lack of efficacy in a dose-finding trial. Neurology 2010;75:619-625.

4. Miller RG, Moore DH, Forshew DA, et al. Phase II screening trial of lithium carbonate in amyotrophic lateral sclerosis: examining a more efficient trial design. Neurology 2011;77:973-979.

5. Wicks P, Vaughan TE, Massagli MP, Heywood J. Accelerated clinical discovery using self-reported patient data collected online and a patient-matching algorithm. Nat Biotechnol 2011;29:411-414.

6. Verstraete E, Veldink JH, Huisman MH, et al. Lithium lacks effect on survival in amyotrophic lateral sclerosis: a phase IIb randomised sequential trial. J Neurol Neurosurg Psychiatry 2012;83:557-564.

7. Al-Chalabi A, Allen C, Counsell C, et al. Lithium in patients with amyotrophic lateral sclerosis (LiCALS): A phase 3 multicentre, randomised, double-blind, placebo-controlled trial. The Lancet Neurology 2013;12:339-345.

Table e-2: Significant predictors for twelve-month survival in the full and genetic datasets.

	**Full dataset (n = 518)**		**Genetic dataset (n = 249)**	
	**Hazard ratio**	**p-value**	**Hazard ratio**	**p-value**
Age (years)	1.04 (95% CI 1.02 – 1.06)	P<0.001	1.05 (95% CI 1.02 – 1.08)	P<0.001
ALSFRS-R slope[a]	0.05 (95% CI 0.02 – 0.12)	P<0.001	0.07 (95% CI 0.02 – 0.26)	P<0.001
VC	0.97 (95% CI 0.96 – 0.98)	P<0.001	0.97 (95% CI 0.95 – 0.99)	P<0.001
UNC13A C/C genotype	-	-	2.39 (95% CI 1.32 – 4.30)	P=0.006
C9orf72 repeat expansion	-	-	2.49 (95% CI 1.17 – 5.32)	P=0.032

VC = upright predicted vital capacity. All predictors for 12-month survival were selected using stepwise backward elimination using the likelihood ratio test.

[a] ALSFRS-R slope was transformed by taking the exponent of the average point loss per month at baseline calculated as $\frac{(\text{ALSFRS−R}_{baseline} - 48)}{Months\ since\ onset}$ and ranges from 0 – 1, where 1 = $e^{-0\ points\ per\ month}$.

Table e-3: Baseline characteristics of the UNC13A carriers per treatment group (n = 46).

	**Placebo** (n = 26)	**Lithium carbonate** (n = 20)	**Standardized difference**
Age at randomization (years)	59 (9)	57 (12)	0.19
Female	11 (42)	11 (55)	0.26
Diagnostic delay (months)	10 (5)	10 (5)	0.16
Disease duration at randomization (months)	18 (9)	17 (9)	0.07
Bulbar site of onset	7 (27)	5 (25)	0.04
Vital capacity (% predicted)	85 (19)	89 (20)	0.21
ALS function rating scale - revised	38 (5)	39 (7)	0.17

Data are mean (SD) or n (%)
