# Supplementary material for: Meta-analysis of pharmacogenetic interactions in amyotrophic lateral sclerosis clinical trials
Source: Neurology. 2017 Oct 31;89(18):1915–22. doi: 10.1212/WNL.0000000000004606 (PMC5664299; doi:10.1212/WNL.0000000000004606)
Supplement: Coinvestigators [file supp_89_18_1915_v2_index.html]

Meta-analysis of pharmacogenetic interactions in amyotrophic lateral sclerosis clinical trials — Meta-analysis of pharmacogenetic interactions in amyotrophic lateral sclerosis clinical trials — Coinvestigators 

# Meta-analysis of pharmacogenetic interactions in amyotrophic lateral sclerosis clinical trials

## Coinvestigators

**Neurology® data supplements are not copyedited before publication. Published editorials and translations have been copyedited.  
 © 2017 American Academy of Neurology.  
  
 Files in this Data Supplement:**

- Coinvestigators - Microsoft Word file
