# Supplementary material for: Meta-analysis of pharmacogenetic interactions in amyotrophic lateral sclerosis clinical trials
Source: Neurology. 2017 Oct 31;89(18):1915–22. doi: 10.1212/WNL.0000000000004606 (PMC5664299; doi:10.1212/WNL.0000000000004606)
Supplement: Coinvestigators [file supp_WNL.0000000000004606_Coinvestigators.docx]

**UKMND-LiCALS Study Group**

*Data monitoring and ethics committee:* C Allen, C Counsell, A Farrin.

*Trial steering committee:* A Al-Chalabi, B Dickie (co-opted observer), J Kelly (co-opted observer), P N Leigh, C L Murphy (co-opted observer), C Payan, G Reynolds, P Shaw, I N Steen, M Thornhill (co-opted observer), J Waters, J Zajicek.

*Trial management group:* P N Leigh, A Al-Chalabi, P J Shaw, C A Young, M Thornhill, I N Steen, C L Murphy.

*Writing committee: Neurosciences Group, School of Clinical and Experimental Medicine, University of Birmingham and Queen Elizabeth Hospital, University Hospitals Birmingham NHS Foundation Trust, Birmingham* K E Morrison (principal investigator), S Dhariwal (research nurse), R Hornabrook (research nurse), L Savage (research nurse); *Institute for Aging and Health, Newcastle University, Newcastle* D J Burn (unmasked physician), T K Khoo (unmasked physician); *King's CTU, King's College London, London* J Kelly (senior data manager), C L Murphy (manager); *King's College London, Institute of Psychiatry, Department of Clinical Neuroscience, London* A Al-Chalabi (chief investigator), A Dougherty (research nurse), P N Leigh (previous chief investigator), L Wijesekera (unmasked physician), M Thornhill (trial manager); *Department of Clinical Neuroscience, King's College Hospital, London* C M Ellis (unmasked physician); *Walton Centre for Neurology and Neurosurgery, Liverpool* R Ali (masked physician), K O'Hanlon (unmasked research nurse), J Panicker (unmasked physician), L Pate (research nurse), P Ray (unmasked physician), L Wyatt (research nurse), C A Young (principal investigator); *Greater Manchester Neurosciences Centre, Salford Royal Hospital and University of Manchester, Manchester* L Copeland (trial administrator), J Ealing (principal investigator), H Hamdalla (masked co-investigator), I Leroi (unmasked physician), C Murphy (unmasked physician), F O'Keeffe (trial administrator), E Oughton (research nurse), L Partington (unmasked research nurse), P Paterson (research nurse), D Rog (unmasked physician), A Sathish (research nurse), D Sexton (research nurse), J Smith (trial administrator), H Vanek (unmasked research nurse); *Department of Neurosciences, The Newcastle Upon Tyne Hospitals NHS Foundation Trust, Newcastle* S Dodds (unmasked research nurse), T L Williams (principal investigator); *Institute of Health and Society, Newcastle University, Newcastle* I N Steen (trial statistician); *The National Hospital for Neurology and Neurosurgery, London* J Clarke (research nurse), C Eziefula (clinical studies officer), R Howard (unmasked physician), R Orrell (principal investigator), K Sidle (unmasked physician), R Sylvester (unmasked physician); *Nuffield Department of Clinical Neurosciences, University of Oxford, Oxford* W Barrett (research nurse), C Merritt (research nurse), K Talbot (principal investigator), M R Turner (unmasked physician), C Whatley (unmasked research nurse), C Williams (unmasked research nurse), J Williams (masked sub-investigator); *Plymouth University Peninsula School of Medicine and Dentistry, Plymouth* C Cosby (clinical trial unit manager), C O Hanemann (principal investigator), I Imam (unmasked physician), C Phillips (clinical studies officer), L Timings (nee Walker; clinical studies officer); *Sheffield Institute for Translational Neuroscience (SITraN), University of Sheffield, Sheffield* S E Crawford (research nurse), C Hewamadduma (masked sub-investigator), R Hibberd (research nurse), H Hollinger (nee Nixon; DeNDRoN motor neuron disease coordinator), C McDermott (unmasked physician), G Mills (research nurse), M Rafiq (masked sub-investigator), P J Shaw (principal investigator), A Taylor (research nurse), E Waines (trial administrator), T Walsh (research nurse); *Department of Neurology, Royal Preston Hospital, Lancashire Teaching Hospitals NHS Foundation Trust, Lancashire* R Addison-Jones (research nurse), J Birt (unmasked research nurse), M Hare (unmasked research nurse), T Majid (principal investigator).

**LitALS Study Group**

R. Tortelli, MD (Università degli Studi di Bari, Site Investigator); E. D'Errico, MD (Università degli Studi di Bari, Site Investigator); I. Bartolomei, MD (Ospedale Bellaria, Bologna, Site Investigator); E. Barbarossa, MD (Ospedale Bellaria, Bologna, Site Investigator); B. Depau, MD (Università degli Studi di Cagliari, Site Investigator); E. Costantino, MD (Università degli Studi di Cagliari, Site Investigator); E. D'Amico, MD (Università degli Studi di Catania, Site Investigator); A. Uncini, MD (Università degli Studi di Chieti, Advisory Committee); C. Manzoli, MD (Università degli Studi di Chieti, Site Investigator); R. Quatrale, MD (Università degli Studi di Ferrara, Site Investigator); E. Sette, MD (Università degli Studi di Ferrara, Site Investigator); E. Montanari, MD (Ospedale di Fidenza, Site Investigator); M. Merello, MD (ASO Sant'Antonio Abate di Gallarate, Site Investigator); D. Zarcone, MD (ASO Sant'Antonio Abate di Gallarate, Site Investigator); M. Mascolo, MD (Università degli Studi di Genova, Site Investigator); M. Vignolo, MD (Università degli Studi di Genova, Site Investigator); S. Messina, MD (IRCCS Istituto Auxologico Italiano, Milan, Site Investigator); C. Morelli, MD (IRCCS Istituto Auxologico Italiano, Milan, Site Investigator); K. Marinou, MD (IRCCS Fondazione Salvatore Maugeri, Milan, Site Investigator); L. Papetti, MD (IRCCS Fondazione Salvatore Maugeri, Milan, Site Investigator); C. Lunetta, MD (NEuroMuscular Omnicentre (NEMO), Fondazione Serena, Milan, Site Investigator); K. Gorni, MD (NEuroMuscular Omnicentre [NEMO], Fondazione Serena, Milan, Site Investigator); D. De Cicco, MD (IRCCS Fondazione Salvatore Maugeri, Mistretta, Site Investigator); C. Pipia, MD (IRCCS Fondazione Salvatore Maugeri, Mistretta, Site Investigator); P. Sola, MD (Università degli Studi di Modena, Site Investigator); E. Georgoulopoulou, MD (Università degli Studi di Modena, Site Investigator); A. Sagnelli, MD (Università degli Studi di Napoli, Site Investigator); G. Tedeschi, MD (Università degli Studi di Napoli, Advisory Committee); G. Oggioni, MD (Università degli Studi del Piemonte Orientale, Novara, Site Investigator); N. Nasuelli, MD (Università degli Studi del Piemonte Orientale, Novara, Site Investigator); C. D'Ascenzo, MD (Università degli Studi di Padova, Site Investigator); V. Cima, MD (Università degli Studi di Padova, Site Investigator); M. Aiello, MD (Università degli Studi di Parma, Site Investigator); R. Rizzi, MD (ASO Arcispedale S. Maria Nuova, Reggio Emilia, Site Investigator); E. Rinaldi, MD (ASO Arcispedale S. Maria Nuova, Reggio Emilia, Site Investigator); M. Luigetti, MD (Università Cattolica del Sacro Cuore, Roma, Site Investigator); A. Conte, MD (Università Cattolica del Sacro Cuore, Roma, Site Investigator); A. Torzini, MD (Università degli Studi di Siena, Site Investigator); G. Greco MD, PhD (Università degli Studi di Siena, Site Investigator); R. Mutani, MD (Università degli Studi di Torino, Advisory Committee); G. Fuda (Università degli Studi di Torino, Site Investigator); M.A. Tommasi, MD (IRCCS Fondazione Salvatore Maugeri, Veruno, Site Investigator).
