# Supplementary material for: Meta-analysis of pharmacogenetic interactions in amyotrophic lateral sclerosis clinical trials
Source: Neurology. 2017 Oct 31;89(18):1915–22. doi: 10.1212/WNL.0000000000004606 (PMC5664299; doi:10.1212/WNL.0000000000004606)
Supplement: Accompanying Editorial [file supp_WNL.0000000000004606_WNL.0000000000004612.pdf]

EDITORIAL

The beginning of precision medicine in ALS?

Treatment to fit the genes

Carmel Armon, MD, MHS
Orla Hardiman, MB, MD, FRCPI, FTCD, MRIA

Correspondence to Dr. Armon: carmel.armon@gmail.com

Neurology® 2017;89:1–2

Amyotrophic lateral sclerosis (ALS) affects approximately 1 in 400 adults of western European ancestry, making it the most common degenerative disease of the motor neuron network. ALS has a mean age at onset of 65 and 85%–90% of cases occur sporadically. Ten to fifteen percent of cases have a recognized genetic contribution, usually in known ALS gene-carrying families.[1] In populations of European extraction, the commonest cause of familial ALS, accounting for up to 40% of familial cases, is the *C9orf72* hexanucleotide repeat expansion.[2] *C9orf72* has a broader associated phenotype including frontotemporal dementia and a more rapid clinical progression. Men with spinal-onset disease have a lower median age at onset and drive the more rapid clinical progression.[2] Other gene variants also associate with earlier age at onset and more rapid progression; for example, the A4V variant mutated *SOD1* gene.[3]

Considerable evidence also supports a genetic contribution to apparently sporadic ALS. Up to 10% of sporadic patients of European descent may have an expanded *C9orf72* hexanucleotide repeat. In addition, number of at-risk variants have been described within different populations. These variants confer only a modest increased risk of developing disease, but influence the phenotype. The single nucleotide polymorphism (SNP) rs12608932 located in the *UNC13A* gene may increase the risk of sporadic ALS by up to 50% in heterozygotes for the C allele of the SNP and by up to 100% in homozygotes.[4] While homozygosity of the C allele has no significant effect on age at onset, it is associated with shortened survival. Approximately 16% of European patients are homozygous for the C allele of rs12608932. These observations underscore the genetic heterogeneity of ALS and raise the question "Does this heterogeneity affect response to disease-modifying treatment?"

In this issue of *Neurology*®, van Eijk et al.[5] reanalyzed data from 3 clinical trials of lithium in patients with ALS, which showed no overall treatment effect, to evaluate the possibility that there might nevertheless be a genetically defined subgroup of patients who demonstrate a treatment effect. They found that 12-month survival for the subgroup homozygous for the C allele of rs12608932 improved from 40% to 70% with lithium treatment. Due to small numbers (20 patients in the treated group and 26 controls), the 95% confidence intervals of these point observations were wide, and overlapped ($p = 0.056$). However, treatment effect remained after adjustment for baseline imbalances (vital capacity, sex), age, and Amyotrophic Lateral Sclerosis Functional Rating Scale–revised slopes ($p = 0.04$). Only those harboring the risk allele of *UNC13A* showed this improved survival; in fact, those who were not rs12608932 C allele homozygotes had slightly worse survival when treated with lithium.

Even though patients with the *C9orf72* variant had similar survival to the *UNC13A* C allele homozygotes, no treatment effect was seen for the *C9orf72* patients. The authors thereby established that treatment effect depended on the specific mechanisms that mediated accelerated progression in the responding group, and not on the accelerated course itself. If upheld by a confirmatory study, as suggested by the authors, this is a major accomplishment that brings treatment of ALS into the realm of a precision medicine. We concur that the findings from this post hoc subgroup analysis require replication in a prospective randomized, double-blinded, controlled trial with parallel placebo controls. Prospective confirmation of the findings will set aside critiques of the present report reflecting methodologic issues, such as an imbalance between treatment arms on prognostic factors, early deaths among controls, multiple comparisons, and publication bias. The authors calculated that it might be necessary to screen up to 1100 patients with ALS (PALS) to find the 140 rs12608932 C allele homozygous PALS needed for this confirmatory trial. We support this effort because, if confirmed, it may offer a simple, inexpensive intervention to 1 in 6 PALS that increases their chances of survival at 1 year by 75%.

These findings also have implications for the design of future clinical trials. Confirmation that a genetic subgroup may respond to treatment, while other PALS do not, or may be made worse, means

See page 1915

From the Tel Aviv University Sackler School of Medicine and Yitzhak Shamir (Assaf Harofeh) Medical Center (C.A.), Israel; and Academic Unit of Neurology (O.H.), Trinity Biomedical Sciences Institute, Trinity College, Dublin, Ireland.

Go to Neurology.org for full disclosures. Funding information and disclosures deemed relevant by the authors, if any, are provided at the end of the editorial.

© 2017 American Academy of Neurology. Unauthorized reproduction of this article is prohibited.

that future clinical trials in PALS should attempt to capture complete genetic information regarding participants, to look proactively for these possibilities. While it may not be possible to power studies to detect every gene-by-treatment interaction, large effect sizes occurring in a reasonably large minority (10%–20%) of PALS may come to attention, and be subjected to confirmatory studies. These findings also raise the important question as to the validity of the current models of drug development in ALS, which continue to rely on the mutant SOD1 transgenic rodent model.[6] While this model has served the field well in understanding the biological pathways implicated in mSOD1 ALS, the complexity of non-mSOD1 human disease will require a more nuanced approach. Genomic profiling remains in its infancy in ALS. However, the work of van Eijk et al.[5] marks the end of the beginning. The novel insights open a new chapter and provide new impetus to the field in its search for a cure.

STUDY FUNDING

No targeted funding reported.

DISCLOSURE

The authors report no disclosures relevant to the manuscript. Go to Neurology.org for full disclosures.

REFERENCES

1. Armon C. Accrued somatic mutations (nucleic acid changes) trigger ALS: 2005–2015 update. Muscle Nerve 2016;53:842–849.
2. Rooney J, Fogh I, Westeneng HJ, et al. C9orf72 expansion differentially affects males with spinal onset amyotrophic lateral sclerosis. J Neurol Neurosurg Psychiatry 2017;88: 281.
3. Andersen PM, Nilsson P, Keränen ML, et al. Phenotypic heterogeneity in motor neuron disease patients with CuZn-superoxide dismutase mutations in Scandinavia. Brain 1997;120:1723–1737.
4. Vidal-Taboada JM, Lopez-Lopez A, Salvado M, et al. UNC13A confers risk for sporadic ALS and influences survival in a Spanish cohort. J Neurol 2015;262:2285–2292.
5. van Eijk RPA, Jones AR, Sproviero W, et al. Meta-analysis of pharmacogenetic interactions in amyotrophic lateral sclerosis clinical trials. Neurology 2017;89:1915–1922.
6. Mitsumoto H, Brooks BR, Silani V. Clinical trials in amyotrophic lateral sclerosis: why so many negative trials and how can trials be improved? Lancet Neurol 2014;13: 1127–1138.

© 2017 American Academy of Neurology. Unauthorized reproduction of this article is prohibited.
